# Statistical Methods and Machine Learning Algorithms for Investigating Metabolic Syndrome in Temporomandibular Disorders: A Nationwide Study

**DOI:** 10.3390/bioengineering11020134

**Published:** 2024-01-29

**Authors:** Harry Chweidan, Nikolay Rudyuk, Dorit Tzur, Chen Goldstein, Galit Almoznino

**Affiliations:** 1 Department of Prosthodontics, Oral and Maxillofacial Center, Israel Defense Forces, Medical Corps, Tel-Hashomer, Ramat Gan 02149, Israel; 2 Medical Information Department, General Surgeon Headquarters, Israel Defense Forces, Medical Corps, Tel-Hashomer, Ramat Gan 02149, Israel; 3 Big Biomedical Data Research Laboratory, Dean’s Office, Hadassah Medical Center, Faculty of Dental Medicine, Hebrew University of Jerusalem, Jerusalem 91120, Israel; 4 Department of Oral Medicine, Sedation & Maxillofacial Imaging, Hadassah Medical Center, Faculty of Dental Medicine, Hebrew University of Jerusalem, Jerusalem 91120, Israel

**Keywords:** temporomandibular disorders (TMDs), metabolic syndrome (MetS), anemia, hypertension, machine learning, algorithm, big data analysis, computational analysis

## Abstract

The objective of this study was to analyze the associations between temporomandibular disorders (TMDs) and metabolic syndrome (MetS) components, consequences, and related conditions. This research analyzed data from the Dental, Oral, Medical Epidemiological (DOME) records-based study which integrated comprehensive socio-demographic, medical, and dental databases from a nationwide sample of dental attendees aged 18–50 years at military dental clinics for 1 year. Statistical and machine learning models were performed with TMDs as the dependent variable. The independent variables included age, sex, smoking, each of the MetS components, and consequences and related conditions, including hypertension, hyperlipidemia, diabetes, impaired glucose tolerance (IGT), obesity, cardiac disease, obstructive sleep apnea (OSA), nonalcoholic fatty liver disease (NAFLD), transient ischemic attack (TIA), stroke, deep venous thrombosis (DVT), and anemia. The study included 132,529 subjects, of which 1899 (1.43%) had been diagnosed with TMDs. The following parameters retained a statistically significant positive association with TMDs in the multivariable binary logistic regression analysis: female sex [OR = 2.65 (2.41–2.93)], anemia [OR = 1.69 (1.48–1.93)], and age [OR = 1.07 (1.06–1.08)]. Features importance generated by the XGBoost machine learning algorithm ranked the significance of the features with TMDs (the target variable) as follows: sex was ranked first followed by age (second), anemia (third), hypertension (fourth), and smoking (fifth). Metabolic morbidity and anemia should be included in the systemic evaluation of TMD patients.

## 1. Introduction

Temporomandibular disorders (TMDs) represent a comprehensive classification encompassing diverse clinical manifestations that result in anomalous, deficient, or compromised functioning of the temporomandibular joint(s) (TMJ) and the associated masticatory muscles [1].

TMDs constitute the predominant source of non-dental chronic painful conditions within the orofacial domain, and stand second in the prevalence of musculoskeletal ailments that causes pain and impairment, following chronic lower back pain, impacting up to 12% of the populace [2].

The suffering of patients is manifested in different intensities of pain and discomfort during function to the point of difficulty in eating or speaking. Thus, TMDs may negatively influence routine tasks, social conduct, psycho-emotional conditions, and life quality [3]. These consequences lead to increased healthcare use and social costs, with yearly expenditure appraised at USD four billion [2].

The etiology and progression of TMDs are complex and poorly understood, although numerous etiologic factors that can initiate or contribute to TMDs have been identified. Among the primary factors that have been identified to contribute to the development of TMDs are trauma, psychological distress, increased pain sensitivity, and activities related to parafunction, including bruxism and clenching [4,5]. Furthermore, recent genetic research suggests that variations in the genetic profile of people could potentially exert a significant impact on the perception of pain, thus affecting the susceptibility to develop TMDs [6].

Associations with inflammatory conditions, as well as insufficient nutrient levels among individuals diagnosed with TMDs, have been documented [7].

Prior research analyzed the correlation between TMDs and separately taken component constituents of metabolic syndrome (MetS) [8,9]. MetS, colloquially referred to as ‘Syndrome X’, is a cluster that encompasses a set of interconnected conditions, including central obesity, dyslipidemia, insulin resistance, and hypertension [10]. These conditions collectively amplify susceptibility to cardiovascular diseases and the onset of type 2 diabetes mellitus [10]. Additionally, there is documented evidence linking the syndrome with nonalcoholic fatty liver disease (NAFLD) [11] and obstructive sleep apnea (OSA) [12]. MetS stands as a highly prevalent global health concern, affecting a substantial proportion of the adult population (estimated to be around 20–25% worldwide), meaning that every fourth person in the adult population suffers from MetS [10]. Over the past two decades, various definitions of MetS have emerged, characterized by consensus regarding its key components but discrepancies in the recommended diagnostic criteria [13,14]. It is widely acknowledged that the presence of any MetS component serves as a pivotal signal for the comprehensive assessment of other associated risk factors [13].

While previous studies investigated the association between TMDs and individual components of MetS, to the utmost extent of our cognizance, there are no big data studies utilizing statistical and machine learning (ML) models present in the English literature that study the associations between TMDs, MetS cluster constituents, their repercussions, and associated disorders, including biochemistry test results within the demographic of individuals ranging from youth to middle adulthood.

In recent times, ML has assumed a pivotal role across diverse domains, with its notable impact extending into the medical arena [15,16]. This influence stems from ML’s adeptness at discerning intricate patterns and extracting insights from convoluted datasets. Notably, graph-based deep learning has been employed for medical diagnostic purposes [17], while inverse reinforcement learning (IRL) algorithms have demonstrated efficacy in optimizing performance within intricate systems [18]. The progress witnessed in ML exhibits promise in a myriad of medical applications [19,20,21]. The potential ramifications of ML are particularly salient in advancing the comprehension and treatment of intricate medical conditions such as TMDs.

The predominant consensus asserts that inflammation processes play a role in MetS pathogenesis [22], and thus the amalgamation of biochemistry test outcomes is profoundly important. This multifaceted strategy enriches the comprehensiveness and depth of knowledge of our understanding of biological systems, particularly in the evaluation of hyperglycemia (glycated hemoglobin, fasting glucose), lipid profiles (cholesterol, triglycerides, lipoproteins), and markers of inflammation such as C-reactive protein (CRP).

Considering the above-mentioned unmet needs, our primary aim was to investigate the associations between TMDs and the following parameters: (a) diagnoses related to MetS, and (b) ancillary diagnostic tests, including biochemistry blood assessments utilized in the evaluation of MetS constituents. The hypothesis of this study posited a discernible association between TMDs and certain MetS components. For the exploration of these connections, this study will employ a novel combination of statistical and machine learning (ML) models to enhance comparisons between the models and to validate the findings. By addressing these aims of the study, our objective is to advance TMD and MetS research and shape new avenues for future research and clinical applications that hold critical relevance for policymakers in updating protocols and prevailing guidelines.

## 2. Methods

### 2.1. Research Population

This investigation forms a part of the Dental, Oral, Medical Epidemiological (DOME) big data record-based research project [23,24,25,26,27,28,29,30]. Previous publications have extensively utilized and elucidated the DOME initiative, with a singular paper devoted to outlining the procedural framework and research methodologies of the DOME project [23]. The DOME project is a large-scale, systematized, and comprehensive depository that integrates demographic, dental, and medical records within a nationwide population of individuals ranging from youth to middle adulthood from the military who sought routine medical and dental examinations at the Israel Defense Forces (IDF) general and dental clinics [23]. Cross-referencing this demographic, dental, and medical information affords us a unique opportunity to discern associations between TMD diagnosis and MetS-related conditions on an extensive and unparalleled scale.

### 2.2. Research Ethics Clearance

This research adheres to the STROBE guidelines and was approved by the Institutional Review Board (IRB) of the Medical Corps (approval number: IDF-1281-2013). The IRB authorized this study as exempt from the necessity to obtain informed consent due to the study being retrospective and only involving the review of medical information.

### 2.3. Enrollment Criteria

Inclusion Criteria: This cross-sectional study considered male and female individuals aged 18 to 50 years who were affiliated with the Israel Defense Forces (IDF) and sought dental care at IDF dental clinics during the period from 1 January 2015 to 1 January 2016, and for whom comprehensive socio-demographic, medical, and dental records were available.

Criteria for exclusion: Participants with incomplete data records within the specified data sources were not included in the research.

### 2.4. Information Acquisition

The IDF Medical Information Department furnished the data sourced simultaneously from three IDF electronic systems, specifically, dental patient records (DPRs), medical patient records (computerized patient records (CPRs)), and the socio-demographic electronic systems housing the socio-demographic characteristics of individuals from the military, as elaborated upon in our previous publication [28]. The process of data mining was executed in an anonymized manner by the Medical Corps’ Department of Medical Information, as described previously [23].

### 2.5. Definitions of Study Variables

Demographic and smoking status variables: Sex: male/female; age in years; current smoker: yes/no.

Medical diagnoses: The computerized patient record (CPR) repository utilizes the International Classification of Diseases, 9th Revision, Clinical Modification (ICD-9-CM), as the foundation for diagnostic purposes.

1. The dependent variable: Temporomandibular joint disorders (TMDs), based on the 2015 ICD-9-CM Diagnosis Code 524.60, Temporomandibular joint disorders, unspecified.
2. Systemic comorbidities linked to Metabolic Syndrome (MetS) were incorporated within the study as independent variables defined according to the ICD-9-CM diagnostic criteria. These diagnoses are depicted in Table 1 in the results section.

Ancillary test findings including biochemistry blood test results: The supplementary test outcomes also sourced from the CPR encompassed an array of assessments utilized for the evaluation of metabolic syndrome (MetS) components, including biochemistry blood laboratory tests. These assessments encompassed, as previously delineated [23,29]. The ancillary tests are depicted in Table 2 in the results section.

### 2.6. Analysis Strategy

An innovative integration of statistical and machine learning (ML) models was utilized for analysis of the data.

#### 2.6.1. Statistical Analysis

Statistical procedures were conducted utilizing SPSS software version 28.0 (IBM, Chicago, IL, USA). Descriptive statistics entailed representing continuous variables through means and standard deviations (SDs), while categorical variables were depicted by frequencies and corresponding percentages.

Bivariate analysis: For the bivariate analysis, we scrutinized the association between temporomandibular joint disorders (TMDs) as the dependent variable and their independent variables. Categorical parameters were assessed through Pearson’s chi-square test or the likelihood ratio test, and continuous variables were analyzed using non-paired *t*-tests for non-paired samples. Odds ratios (ORs) were computed, employing linear regression for continuous variables and binary logistic regression for categorical variables.

Multicollinearity analysis: After the bivariate analyses, multicollinearity assessments were performed using linear regression to evaluate the interrelationships among the independent variables. In cases where substantial collinearity was detected between two variables, only one was incorporated into the model, with the specific variable chosen to be contextually determined. Variance inflation factors (VIFs), calculated as 1 divided by the tolerance, were computed. While VIF values below 10 typically denote collinearity, this study applied a VIF threshold of less than 2.5, due to the potential issue of less robust models.

Multivariable analysis: Subsequent to the bivariate analysis and collinearity evaluation, a multivariable binary logistic regression analysis was executed with TMD as the dependent variable. Independent variables identified as statistically significant in the bivariate analysis that were not marked by high collinearity were incorporated. All associations described were statistically significant at *p* = 0.01.

#### 2.6.2. Machine Learning (ML) Models

In the execution of machine learning (ML) models, we harnessed the Python scikit-learn package [31]. We employed XGBoost, a highly efficient gradient-boosting framework that proves particularly adept for supervised machine learning tasks in the domains of regression and classification [32].

The goal of the ML model was to explore the relative feature significance and generate a prioritized variables list according to their importance in the task of the classification of TMDs as the target variable. The model underwent a rigorous evaluation with a five-fold cross-validation approach [33], deploying distinct training and testing dataset ratios such as train–test partitions of 70–30% and 80–20%. To affirm the robustness of the XGBoost ML model, we additionally conducted two alternative ML models to assess feature importance: Gini Importance [34] and Information Gain based on Entropy [35].

Evaluating Adherence to Reporting Standards in Machine Learning Research: To assess the completeness of this research to the standards of reporting research in the field of machine learning, we utilized the checklist of TRIPOD (Transparent Reporting of a Multivariable Prediction Model for Individual Prognosis or Diagnosis; www.tripod-statement.org, accessed on 5 November 2023) for the validation of the prediction models.

The checklist comprises 20 main elements, accompanied by a cumulative sum of 31 sub-components, that address different elements encompassing the validation of prediction models, including title, abstract, introduction, methodology, results, discussion, and funding disclosures.

Each item was scored as adherent (1) or not adherent (0). Subsequent analysis demonstrated adherence of the research to all TRIPOD elements, with 3 elements identified as non-relevant. The findings related to the TRIPOD elements were precisely articulated, with in-depth documentation of the adherence to the specified TRIPOD elements.

## 3. Results

### 3.1. The Associations of Temporomandibular Disorders (TMDs) with Demographics, Smoking Status, and Systemic Conditions

The prevalence of TMDs in the study population was 1.43% (1899/132,529). Table 1 presents the demographics, smoking status, MetS constituents, their repercussions, and associated disorders of patients with TMDs compared to those without TMDs. A statistically significant positive association was demonstrated between TMDs and the following parameters: S/P (status post) transient ischemic attack (TIA) was associated with over 5-fold odds of having TMDs; obstructive sleep apnea (OSA) and deep venous thrombosis (DVT) had over 4-fold odds; nonalcoholic fatty liver disease (NAFLD), impaired glucose tolerance (IGT), and anemia had over a 3-fold odds; and female sex, smoking, hypertension, hyperlipidemia, type 2 diabetes, obesity, and cardiac disease were associated with over 2-fold odds of having TMDs (Table 1).

**Table 1:** The associations of temporomandibular disorders (TMDs) with demographics, smoking status, and systemic conditions. *: non-paired *t*-test, ^: Pearson chi-square, #: linear regression, ##: binary logistic regression; OR: odds ratio, CI: confidence interval.

Parameter		TMD Mean ± SD	Without TMD Mean ± SD	*p* Value *	OR and 95% CI #
Age		25.72 ± 8.03	21.83 ± 5.97	<0.001	1.07 (1.06–1.07)
Parameter	Variable	TMD No. (%)	Without TMD No. (%)	*p*-Value ^	OR (95% Confidence Interval) ##
Sex	Male	1073 (1.1%)	98,393 (98.9%)	<0.001	1
	Female	826 (2.5%)	32,237 (97.5%)		2.34 (2.14–2.57)
Smoking	Yes	223 (3.2)	6661 (96.8)	<0.001	2.47 (2.15–2.85)
	No	1676 (1.3)	123,969 (98.7)		1
Hypertension	Yes	77 (2.3)	3286 (97.7)	<0.001	2.19 (1.60–2.98)
	No	1822 (1.4)	127,344 (98.6)		1
Hyperlipidemia	Yes	285 (3.7)	7441 (96.3)	<0.001	2.92 (2.57–3.32)
	No	1614 (1.3)	123,189 (98.7)		1
Type 2 diabetes	Yes	10 (2.9)	335 (97.1)	0.022	2.06 (1.09–3.86)
	No	1889 (1.4)	130,295 (98.6%)		1
Impaired glucose tolerance (IGT)	Yes	6 (4.7)	122 (95.3)	0.002	3.39 (1.49–7.70)
	No	1893 (1.4)	130,508 (98.6)		1
Obesity	Yes	253 (3.4)	7195 (96.6)	<0.001	2.63 (2.30–3.01)
	No	1646 (1.3)	123,435 (98.7)		1
Nonalcoholic fatty liver disease (NAFLD)	Yes	43 (4.6)	895 (95.4)	<0.001	3.35 (2.46–4.57)
	No	1856 (1.4)	129,735 (98.6)		1
Obstructive sleep apnea (OSA)	Yes	18 (5.7)	300 (94.3)	<0.001	4.15 (2.57–6.70)
	No	1881 (1.4)	130,330 (98.6)		1
Cardiac disease	Yes	110 (3.1)	3488 (96.9)	<0.001	2.24 (1.84–2.72)
	No	1789 (1.4)	127,142 (98.6)		1
S/P Transient ischemic attack (TIA)	Yes	7 (7.1)	92 (92.9)	<0.001	5.25 (2.43–11.33)
	No	1892 (1.4)	130,538 (98.6)		1
S/P Stroke	Yes	6 (6.5)	86 (93.5)	<0.001	4.81 (2.10–11.02)
	No	1893 (1.4)	130,544 (98.6)		1
S/P Deep venous thrombosis (DVT)	Yes	7 (6.5)	101 (93.5)	<0.001	4.78 (2.22–10.30)
	No	1892 (1.4)	130,529 (98.6)		1
Anemia	Yes	320 (4.1)	7440 (95.9)	<0.001	3.35 (2.97–3.79)
	No	1579 (1.3)	123,190 (98.7)		1

### 3.2. The Associations of Temporomandibular Disorders (TMDs) with Ancillary Test Findings including Biochemistry Blood Test Results Used in the Workup of MetS Components

The associations of TMDs with ancillary test findings, including laboratory biochemistry assays employed in the work-up of MetS components, are depicted in Table 2. There was a statistically significant positive association between TMD and body mass index (BMI), cholesterol, and high-density lipoprotein (HDL). Nevertheless, the associations were weak with odds ratios (ORs) closely approximating a value of 1 (for this reason, three decimal places are displayed in Table 2). Moreover, the rest of the ancillary tests presented in Table 2 had no statistically significant associations with TMDs.

**Table 2:** The associations of temporomandibular disorders (TMDs) with ancillary test findings including biochemistry blood test results used in the workup of MetS components. *: non-paired *t*-test, #: linear regression; OR: odds ratio, CI: confidence interval. Statistically significant results are in bold.

Parameter	TMD		Without TMD		*p* Value *	OR and 95% CI #
	N	Mean ± SD	N	Mean ± SD		
Weight (kilograms)	1104	73.02 ± 28.43	65,513	73.30 ± 32.44	0.778	1.000 (0.998–1.002)
Body mass index (BMI)	1100	24.76 ± 4.74	65,294	24.26 ± 4.29	**0.001**	1.026 (1.012–1.039)
C-reactive protein (CRP) (mg/L)	826	3.96 ± 6.85	29,529	3.76 ± 10.26	0.571	1.002 (0.996–1.008)
Glycated hemoglobin (HbA1c) (%)	69	5.36 ± 0.94	1874	5.40 ± 0.97	0.761	0.960 (0.738–1.249)
Fasting glucose (mg/dL)	70	86.75 ± 9.92	2457	87.13 ± 11.99	0.754	0.997 (0.977–1.018)
Cholesterol (mg/dL)	867	178.89 ± 33.47	27,313	175.72 ± 35.69	**0.006**	1.002 (1.001–1.004)
High-density lipoprotein (HDL) (mg/dL)	867	50.06 ± 12.89	27,306	48.22 ± 11.73	**<0.001**	1.013 (1.007–1.018)
Low-density lipoprotein (LDL) (mg/dL)	685	109.31 ± 27.83	19,528	108.31 ± 30.11	0.354	1.001 (0.999–1.004)
LDL cholesterol calculated (mg/dL)	565	110.06 ± 28.07	16,893	108.32 ± 30.48	0.147	1.002 (0.944–1.010)
Triglycerides (mg/dL)	867	104.87 ± 67.46	27,316	104.45 ± 63.92	0.851	1.000 (0.999–1.001)
Very-low-density lipoprotein (VLDL) (mg/dL)	866	20.52 ± 11.08	27,265	20.61 ± 11.20	0.817	0.999 (0.993–1.005)
Non-HDL cholesterol (mg/dL)	561	130.99 ± 32.22	16,261	129.45 ± 35.10	0.270	1.001 (0.999–1.004)

### 3.3. Multivariable Analysis and Collinearity Statistics Evaluating Temporomandibular Disorders (TMDs) as a Dependent Variable with Significantly Associated Parameters Identified in the Bivariate Analysis

After conducting bivariate analyses, a linear regression analysis was carried out to determine the collinearity among the independent variables that exhibited statistical significance. The collinearity statistics results are presented in Table 3 and rule out collinearity, since all VIF values are below 2.5.

Following that, we carried out a multivariable binary logistic regression analysis with TMD diagnosis as the dependent variable, which is also presented in Table 3. The multivariable analysis included statistically significant independent variables following the bivariate analysis, and did not exhibit collinearity. All independent variables were collectively incorporated in a single step within the multivariable analysis. Female sex was associated with over 2.5-fold odds of having TMD, and anemias was associated with over 1.5-fold odds and the ORs for age were close to 1 (Table 3).

**Table 3:** Multivariable analysis and collinearity statistics with temporomandibular disorders (TMDs) as a dependent variable with statistically significant parameters in the bivariate analysis. SE: standard error; VIF: variance inflation factor; statistically significant values are in bold.

Parameter	Multivariable Binary Logistic Regression Analysis				Collinearity Statistics Using Linear Regression Analysis	
	B	SE	*p* Value	OR (95% CI)	Tolerance	VIF
(Intercept)	5.31	0.08		0.005 (0.004–0.006)		
Age	0.07	0.003	**<0.001**	1.07 (1.06–1.08)	0.485	2.060
Sex: women vs. men	0.97	0.05	**<0.001**	2.65 (2.41–2.93)	0.939	1.065
Smoking	0.07	0.08	0.383	1.07 (0.91–1.26)	0.775	1.291
Hypertension	0.21	0.17	0.233	1.23 (0.87–1.73)	0.908	1.102
Hyperlipidemia	0.68	0.08	0.448	1.07 (0.89–1.27)	0.558	1.791
Type 2 diabetes	0.23	0.24	0.344	1.26 (0.78–2.04)	0.928	1.078
Impaired glucose tolerance (IGT)	0.05	0.46	0.910	1.05 (0.42–2.63)	0.968	1.033
Obesity	0.06	0.08	0.429	1.07 (0.90–1.26)	0.681	1.468
Cardiac disease	0.11	0.10	0.305	1.11 (0.90–1.38)	0.938	1.066
Obstructive sleep apnea (OSA)	0.49	0.25	0.051	1.63 (0.99–2.66)	0.979	1.022
Nonalcoholic fatty liver disease (NAFLD)	0.31	0.17	0.069	1.37 (0.97–1.93)	0.903	1.107
S/P transient ischemic attack (TIA)	0.36	0.42	0.385	1.43 (0.63–3.27)	0.960	1.041
S/P stroke	0.21	0.49	0.674	1.23 (0.46–3.26)	0.962	1.039
S/P deep venous thrombosis (DVT)	0.61	0.40	0.131	1.83 (0.83–4.04)	0.996	1.004
Anemia	0.52	0.06	**<0.001**	1.69 (1.48–1.93)	0.917	1.090

Following the multivariable analysis, we performed a second multivariate logistic regression analysis stratified according to age, which is presented in Table 4. We split the total sample into two groups: 18–30 years (119,579 patients, 90.2%) and 31–50 years (12,949, 9.8%). In the younger age group (18–30 years) the significant parameters were sex, smoking, hyperlipidemia, obesity, cardiac disease, OSA, and anemia. In the older age group (31–50 years), the significant parameters were sex, NAFLD, and anemia. Sex and anemia were the only parameters that retained a statistically significant association in both age groups.

### 3.4. Feature Importance Based on XGBoost Machine Learning (ML) Algorithm with Temporomandibular Disorders (TMDs) as a Target Variable

**Figure 1:**
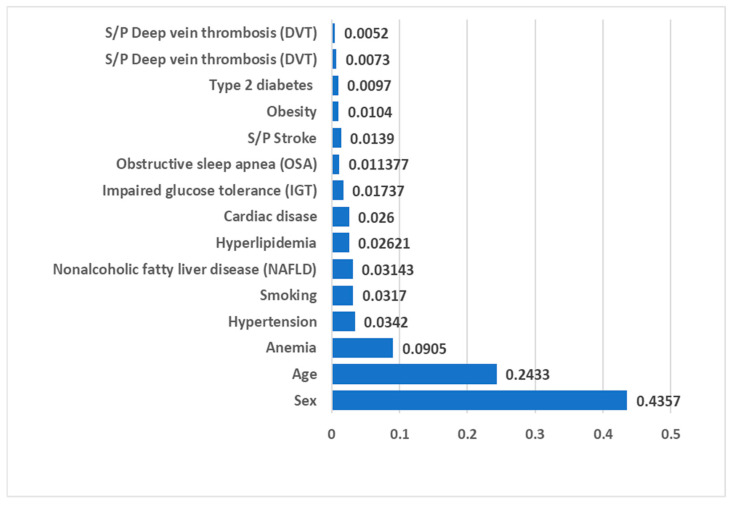
Feature importance scores produced by XGBoost algorithm for TMD diagnosis as a target variable.

Both the Gini Importance and Information Gain based on Entropy methods yielded results for model fitness measurements, including metrics like the area under the curve (AUC) and accuracy, which exhibited a high degree of similarity to the outcomes obtained with the XGBoost model. Therefore, we present the results of the XGboost ML algorithm in Figure 1. The AUC was 0.748, recall score = 0.703, precision = 0.027, and accuracy was 0.660. The thresholds indicating excellent discrimination in the AUC results lie within the range of 0.7 to 0.8 [36]. Furthermore, the XGBoost model demonstrates a nearly two-fold increase in precision (2.7%) compared to the TMD prevalence in the study population (1.43%), showcasing its proficiency in precise disease detection while effectively reducing false positive results. The feature importance scores derived from the XGBoost algorithm, as illustrated in Figure 1, reveal the model’s prioritization of feature significance with TMDs (the target variable) as follows: sex holds the top position, followed by age (second), anemia (third), hypertension (fourth), and smoking (fifth).

## 4. Discussion

To the utmost extent of our cognizance, this marks the inaugural research within the English literature that employs novel methods of statistical and ML analytics in a big data context to investigate the association between TMDs and MetS among 132,529 individuals ranging from youth to middle adulthood, using a holistic approach that cross-referenced demographic and medical data including laboratory biochemistry tests at an unmatched scale.

The fusion of clinical data with biochemistry test results empowers investigators to unveil intricate associations between molecular events and systemic responses.

Artificial intelligence (AI) algorithms have been employed in the diagnosis of TMDs. Nonetheless, investigations have employed disparate criteria for patient selection, diverse categorizations of disease subtypes, distinct input data, and varied outcome measures, and consequently, the efficacy of AI models exhibits variability across these studies [37]. For this reason, this study employed a hybrid analytical framework combing both statistical and ML approaches.

Regarding demographic parameters, the outcomes of the investigation align with the literature. Our study population included subjects aged 18–50 years, and similarly, Yap et al. found that TMD signs and symptoms are more predominant among adults between 20 and 40 years of age [38]. Coinciding with our findings of a positive association between TMDs and age in both statistical and ML models, a large prospective clinical trial study representing 2737 TMD subjects also showed an increased prevalence in accordance with age. The annual increment varies, ranging from 2.5% for individuals aged 18 to 24 to 4.5% for those within the age bracket of 35 to 44 [39].

In the present study, we addressed the confounding effect of age by performing a multivariable analysis with age as a continuous parameter, and also stratified our data according to age (18–30 vs. 31–50 years). The younger age group comprised most of the sample (90.2%), and therefore our conclusions should be based on careful interpretation of all analyses performed. Sex and anemia were significant in both age groups, as well as in the multivariable analysis that used age as a continuous variable, and in the ML algorithm, highlighting these factors as the most significant.

In accordance with our observations of a positive association between TMDs and female sex in both statistical and ML models, there is consensus in the existing medical literature that TMDs predominantly affect women [40,41,42]. The results of a US National Health Interview Survey showed that women reported pain in the jaw joint and/or facial pain 2.1 times more often than men [43]. TMD pain was additionally prevalent among women compared to men in studies from Sweden [44] and Finland [45].

In this study, while a significant positive association was found between TMDs and smoking habits in the bivariate analysis, this association was lost following the multivariable analysis, and smoking was ranked only in fifth place by the ML model. The results in the literature are contradictory. Some studies demonstrated that smoking cigarettes was related to both significantly greater TMD pain intensity [46,47,48] and TMJ sounds [46], and to poorer response to treatment than nonsmokers [48]. Contradictory to these results, Wänman et al. found that the manifestation or progression of signs and symptoms of TMDs is not associated with smoking [49]. Yekkalam et al. also found no correlation between smoking and craniomandibular disorders in an adult population [50].

This study aimed to perform an analysis of the association between TMDs and MetS-associated disorders utilizing statistical and ML analytics. The study demonstrated that TMDs are positively associated with systemic conditions related to MetS, and in particular with anemia and hypertension. Anemia maintained a statistically significant association with TMDs after multivariable analysis and emerged as the most highly ranked systemic condition in the ML model (ranked third after sex and age). Corresponding to our results, other studies also found an association between TMDs and anemia. For example, Ohrbach and colleagues identified a noteworthy association between TMDs and different hematologic disorders including anemia, disorders of bleeding, and leukemia, using data from the OPPERA case–control study [8].

Mehra et al. found serum nutrient deficiencies, including anemia, in patients with complex TMDs who underwent surgical joint reconstruction [51]. Orhan et al. found that individuals experiencing persistent anemia exhibited reduced signal intensity in the mandibular condyle bone marrow and posterior band compared to their healthy counterparts, suggesting that anemia might induce modifications in bone marrow without any concurrent internal derangement [52].

A separate investigation exploring tissue oxygen saturation and alterations in oxygenated hemoglobin, deoxygenated hemoglobin, and total hemoglobin within the masseter muscle revealed that subjects with a predisposition to TMDs exhibit irregularities in the deoxygenation of the masseter [53]. Conversely, Staniszewski et al., in a controlled cross-sectional study of 60 TMD patients and 60 healthy controls, failed to establish a correlation between severe systemic illness, malnutrition, and systemic inflammation with TMDs [7].

The association between TMDs and anemia may signify variations in hemoglobin levels concerning age, sex, and smoking habits. For this reason, we used statistical and ML multivariable models that adjusted for these parameters and demonstrated an association between TMDs and anemia, independent of age, sex, and smoking. Another explanation for a positive association between TMDs and anemia was suggested by Mehra et al., who attributed anemia to the deficiency state due to inadequate nutritional intake and utilization dysfunction in TMD patients [51]. An additional compelling rationale for the observed association between TMDs and anemia pertains to the presence of *anemia of inflammation*, also recognized as *anemia of chronic disease*. This form of anemia is common in patients with illness causing protracted immune activation, such as infections, autoimmune disorders, and malignancies [54]. This category has expanded over the years to encompass the effects of aging, obesity, type 2 diabetes, pulmonary arterial hypertension, chronic liver disease, and advanced atherosclerosis, with ramifications of stroke and coronary artery disease [54]. Indeed, in the current study, TMD patients exhibited a higher prevalence of systemic conditions related to MetS compared to those without TMDs.

The highest-ranked MetS-related condition was hypertension, which was ranked fourth by the ML model, although it did not retain a statistical significance with TMDs in the multivariable statistical analysis. Thus, the present research emphasizes the importance of using ML models in addition to classical statistical models, as they can add value to feature importance identification. While there were previous studies that found no correlation between TMDs and hypertension [55,56], there were other studies similar to our findings, such as Sanders et al., who demonstrated an association between the incidence of first onset TMD and increased mean baseline arterial pressure, as well as OSA [57]. Maixner et al. found that painful TMDs exhibit heightened sensitivity to painful stimuli, and may result from dysfunction in the central pain modulatory mechanisms which, in turn, can be influenced by baseline arterial blood pressure [58].

Following hypertension, the next ranked MetS-related condition by the ML algorithm was NAFLD, which was ranked sixth in the feature importance for the task of TMD classification. In recent years, NAFLD has been recognized to be the hepatic manifestation of MetS, and is also termed “metabolic dysfunction-associated fatty liver disease” [11]. While other MetS-related conditions were studied in the context of TMDs, based on a performed literature review, no studies were found regarding the association between TMDs and NAFLD, highlighting the importance of the current study’s holistic approach in analyzing MetS related conditions in the context of TMDs.

Moreover, the holistic approach employed in this investigation enriched the comprehensiveness and depth of our knowledge on molecular biology in the assessment of hyperlipidemia and serum lipid profile parameters (cholesterol, HDL, LDL, non-HDL, triglycerides). Hyperlipidemia was only ranked seventh by the ML algorithm, and while serum cholesterol and HDL were positively associated with TMD, these parameters exhibited ORs close to 1, indicating a weak association. In accordance with our observations, a long-term cohort study found no correlation between TMDs and hyperlipidemia/dyslipidemia [56,59], and another study found no significant association between levels of total cholesterol and TMDs [55].

Furthermore, the multifarious approach included the evaluation of IGT, diabetes, and hyperglycemia (serum glycated hemoglobin, fasting glucose). The ML algorithm ranked IGT and diabetes only in the 9th and 13th places, respectively, and serum glycated hemoglobin and fasting glucose had no significant association with TMDs. Parallel to our investigations, previous studies did not demonstrate a correlation between diabetes and painful TMDs [9,59,60], and while we found no differences in fasting glucose levels, Byun et al. even reported that TMD patients have lower mean levels of fasting blood glucose [55].

Another important biochemical marker incorporated in the analysis was serum CRP. CRP is acknowledged as a significant indicator of persistent inflammatory processes and as one of the major proteins of the acute phase reaction [61]. In the present study, CRP levels were not significantly different between those with and without TMDs, like reports in previous studies [7,62].

The study also analyzed the associations between weight, BMI data, and obesity. A sequence of cross-sectional surveys concluded that BMI does not mirror equivalent adjustments of weight relative to height across different genders or across age cohorts [63].

For this reason, we decided to examine both weight and BMI separately. Obesity did not maintain a statistically significant association with TMDs following multivariable analysis and was ranked 12th in feature importance for TMD classification by the ML algorithm. Our findings demonstrate that weight had no statistically significant association with TMDs, and BMI—although positively associated with TMDs—exhibited ORs close to 1, indicating a weak association. Coinciding with our findings, Jordani et al. demonstrated that painful TMDs exhibited a notable correlation with total body fat percentage, but in the multivariable analysis, obesity did not maintain its significance [64]. Two recent systematic reviews and meta-analyses did not show a clear association between obesity and TMDs and concluded that obesity is not a risk factor for TMDs [65,66].

Consequences of MetS such as cardiac disease, TIA, stroke, and DVT were also taken into consideration in our holistic approach using statistical and ML analyses. These parameters did not retain a statistically significant positive association in the multivariable analysis and were ranked relatively low by the ML algorithm in terms of feature importance during the task of TMD classification. While we were looking at TMD patients, other studies focused on the prevalence of TMD dysfunction among stroke patients and found it was higher compared with the healthy group [60]. However, corresponding to our study, in a nationwide population-based cohort study, Lee et al. also found no association between TMDs and stroke [56]. Another study, researching potential risk factors for chronic TMDs found no correlation of TMDs to different cardiovascular conditions, including mitral valve prolapse, high blood pressure, angina, heart attack, heart failure, and stroke [8].

The principal strengths inherent in the current investigation include a substantial sample size and meticulous adherence to a rigorous protocol that incorporated demographic and medical databases. This enabled us to cross-reference TMD diagnosis with demographic and medical data on an unprecedented scale. Definitions were uniform for all patients. To reduce recall bias, the study used demographic data, medical diagnoses, and medical indexes that were extracted from records devoid of dependence on patient self-reports, except for smoking (which was derived from the records but relied on the reports of the patients). Because of the large dataset, a concern is finding significant but clinically meaningless associations. Therefore, the study employed a rigorous multi-step analytical approach by setting the cut-off for statistical significance at *p* = 0.01, performing collinearity statistics with a VIF cut-off of 2.5 to address the potential pitfalls of variable intercorrelation, and including in the final multivariable model only parameters that exhibited statistical significance in the bivariate analysis, while also demonstrating low collinearity. This meticulous approach accounts for confounding effects, reducing the inflation of type I error rates and enhancing the validity of the results. Moreover, the study utilized a novel approach that combined statistical and ML models to enhance the validity of the findings.

The main limitation of the current research is the cross-sectional study design, which prevents the establishment of causality. Although we used a nationwide military population, further investigations—encompassing extended longitudinal population-based epidemiological surveys conducted in diverse settings and among varied populations—would contribute to the augmentation of generalizability and account for these limitations. Future studies should be multi-centered and should include advanced statistical and artificial intelligence approaches. Federated learning can be employed, which will enable model training across servers holding local data samples without the need to exchange them, thus mitigating concerns related to data security and privacy. Furthermore, alternative forms of data, including textual electronic medical data, vocal information, and auditory data, should be employed in future TMD research.

## 5. Conclusions

This study utilized novel methods of statistical and ML analytics to identify the association between TMDs and MetS among 132,529 individuals ranging from youth to middle adulthood, employing a holistic approach that analyzed demographic, medical, and laboratory data on an unmatched scale. We established that a profile of a “patient that is vulnerable to have TMD” includes the following: female sex, older age, and the presence of anemia and hypertension. In the systemic assessment of patients with TMDs, it is imperative to incorporate examinations for metabolic morbidity and anemia. Evaluating risk factors associated with these conditions is essential for the targeted identification of high-risk populations susceptible to TMDs, MetS, and anemia. Health authorities should be cognizant of these co-morbidities in individuals with TMDs, and facilitate appropriate referrals to both dentists and physicians for comprehensive evaluation.

## Figures and Tables

**Table 4:** Multivariable analysis stratified according to age (18–30 and 31–50 years) with temporomandibular disorders (TMDs) as a dependent variable with statistically significant parameters in the bivariate analysis. SE: standard error; statistically significant values are in bold.

Age Groups	Parameter	Multivariable Binary Logistic Regression Analysis			
		B	SE	*p* Value	OR (95% CI)
Age 18–30	(Intercept)	3.97	0.04	<0.001	0.02 (0.01–0.02)
	Sex: women vs. men	0.88	0.05	**<0.001**	2.41 (2.16–2.68)
	Smoking	0.52	0.14	**<0.001**	1.69 (1.27–2.25)
	Hypertension	0.64	0.28	0.024	1.91 (1.08–3.35)
	Hyperlipidemia	0.49	0.17	**0.005**	1.63 (1.16–2.29)
	Type 2 diabetes	0.31	0.73	0.670	1.36 (0.32–5.74)
	Impaired glucose tolerance (IGT)	0.86	1.06	0.42	2.36 (0.29–19.18)
	Obesity	0.54	0.14	**<0.001**	1.72 (1.31–2.26)
	Cardiac disease	0.42	1.68	**0.01**	1.52 (1.09–2.12)
	Obstructive sleep apnea (OSA)	1.93	0.44	**<0.001**	6.89 (2.88–16.47)
	Nonalcoholic fatty liver disease (NAFLD)	1.13	0.47	0.772	1.14 (0.45–2.91)
	S/P transient ischemic attack (TIA)	0.84	1.04	0.422	2.32 (0.29–18.17)
	S/P stroke	1.12	1.02	0.272	3.09 (0.41–23.15)
	S/P deep venous thrombosis (DVT)	1.08	0.61	0.076	2.94 (0.88–9.75)
	Anemia	0.75	0.08	**<0.001**	2.13 (1.81–2.50)
Age 31–50	(Intercept)	2.89	0.11	<0.001	0.05 (0.04–0.07)
	Sex: women vs. men	0.87	0.11	**<0.001**	2.39 (1.91–3.00)
	Smoking	0.04	0.10	0.679	1.04 (0.85–1.27)
	Hypertension	0.34	0.020	0.142	1.35 (0.90–2.03)
	Hyperlipidemia	0.20	0.10	0.051	1.22 (0.99–1.49)
	Type 2 diabetes	0.07	0.25	0.778	1.07 (0.64–1.78)
	Impaired glucose tolerance (IGT)	0.09	0.47	0.843	1.09 (0.43–2.77)
	Obesity	0.04	1.05	0.688	1.04 (0.84–1.28)
	Cardiac disease	0.04	0.19	0.688	1.04 (0.84–1.28)
	Obstructive sleep apnea (OSA)	0.32	0.30	0.283	1.38 (0.76–2.51)
	Nonalcoholic fatty liver disease (NAFLD)	0.50	0.18	**0.006**	1.65 (1.15–2.37)
	S/P transient ischemic attack (TIA)	0.50	0.46	0.278	1.65 (0.67–4.08)
	S/P stroke	0.47	0.51	0.355	1.60 (0.59–4.34)
	S/P deep venous thrombosis (DVT)	0.57	0.53	0.278	1.77 (0.63–5.03)
	Anemia	0.40	0.11	**<0.001**	1.49 (1.19–1.86)

## Data Availability

Data are contained within the article.
